# Daurinol blocks breast and lung cancer metastasis and development by inhibition of focal adhesion kinase (FAK)

**DOI:** 10.18632/oncotarget.18983

**Published:** 2017-07-04

**Authors:** Jong Kyu Woo, Hyun Jin Jung, Ji-Youn Park, Ju-Hee Kang, Byung Il Lee, DongYun Shin, Chu Won Nho, Soo-Young Cho, Je Kyung Seong, Seung Hyun Oh

**Affiliations:** ^1^ Gachon Institute of Pharmaceutical Sciences, Gachon University, Incheon, Republic of Korea; ^2^ Korea Mouse Phenotyping Center, College of Veterinary Medicine, Seoul National University, Seoul, Republic of Korea; ^3^ National Cancer Center, Goyang-si, Republic of Korea; ^4^ Korea Institute of Science and Technology (KIST), Gangneung Institute, Gangneung-si, Republic of Korea

**Keywords:** daurinol, breast cancer, lung cancer, FAK, metastasis

## Abstract

FAK overexpression has been reported in diverse primary and metastatic tumor tissues, supporting its pro-tumorigenic and pro-metastatic roles. Therefore, we have developed a neo-treatment strategy using daurinol to effectively treat cancer metastasis. Daurinol blocked cancer cell migration and invasion *in vitro* and exhibited anti-metastatic activity in an experimental metastasis model of breast and lung cancer. Daurinol selectively inhibited phosphorylation of FAK at Tyr925, Tyr576/577, and Tyr397 sites in a dose- and time-dependent manner. Daurinol effectively suppressed migration and invasion of MDA-MB-231 and A549 cancer cells. These data were associated with inhibition of expression and secretion of invasion factors, including matrix metalloproteinase (MMP) 2, MMP9, and urokinase plasminogen activator (uPA). Consistent with these *in vitro* results, daurinol (10 mg/kg; Oral gavage) effectively inhibited breast and lung cancer metastasis in a mouse model. In addition, daurinol showed strong suppressive activity of cell survival as revealed by colony formation assays. Analysis of cellular phenotypes revealed that inhibition of FAK phosphorylation in cancer cells limited colony formation, cell migration, and invasion, thereby reducing the cell proliferation rate. Furthermore, daurinol significantly reduced tumor development in 4-(methylnitrosoamino)-1-(3-pyridyl)-1-butanone (NNK)/benzo(a)pyrene (BaP)-treated A/J mice. Our results suggest that daurinol suppresses lung metastasis through inhibition of migration and survival via blockade of FAK activity.

## INTRODUCTION

Metastasis is the leading cause of cancer-associated mortality between men and women, yet it remains the most poorly understood component of cancer pathogenesis [1, 2]. Cancer treatment becomes difficult when tumor cells metastasize into distant body organs. Therefore, prevention of cancer cell metastasis is an effective strategy for successful management of human cancers. The process of cancer metastasis consists of a long series of sequential, interrelated steps that involve movement of tumor cells from their primary location to a secondary site [1]. Further, cancer metastasis involves the regulated adhesion and invasion of individual cancer cells controlled by key signaling molecules. Among them, FAK has been shown to be positioned at the crossroad between integrin and growth factor receptor signaling and thus control biological processes such as adhesion, migration, differentiation, survival, and angiogenesis [3].

The mechanism of cancer invasion and metastasis is a complicated multistep process involving multiple genetic alterations. Focal Adhesion Kinase (FAK), a 125 kD non-receptor protein tyrosine kinase also known as protein tyrosine kinase2 (PTK2), is located at sites of integrin clustering in focal adhesions and is involved in several cellular functions such as survival, invasion, motility, adhesion, metastasis, proliferation, and angiogenesis [4–10]. FAK is autophosphorylated at Tyr397, which results in high binding affinity for the SH2 domain of src family kinases [11, 12]. FAK is positioned at the intersection of various signaling pathways that promote cancer growth and metastasis. This includes kinase-dependent control of cell motility, invasion, cell survival, and transcriptional events promoting the epithelial-to-mesenchymal transition (EMT) [10, 13–15]. Indeed, FAK is overexpressed in a variety of human tumors, including head and neck, cervical, colorectal, gastric, hepatocellular, lung, breast, prostate, ovarian, pancreatic, and thyroid tumors [16–25]. Targeting FAK is a promising approach to overcome cancer therapy resistance. Several FAK inhibitors are currently under investigation in clinical trials [26].

Daurinol, a bioactive component derived from the *arylnaphthalene lignan* plant, has been widely used in therapeutic preparations for centuries due to its anti-inflammatory, anti-cancer, and chemotherapeutic properties [27]. Anti-cancer activities of daurinol against various human cancer cells have been reported. Recent studies have demonstrated its chemotherapeutic efficacy in colon [28], ovarian [29], and lung cancers [30]. In addition, daurinol has been reported to enhance radiation therapy efficacy in human lung cancers [30]. Especially, its anti-cancer properties have received a great deal of interest. Daurinol has been identified as a drug that interferes with the cell cycle to induce apoptosis. The molecular basis of its anti-cancer activity is attributed to its effects on several targets, including transcription factors, growth regulators, and cell mitosis-regulating molecules. However, the underlying molecular mechanisms of daurinol are still under investigation, especially regarding its anti-metastatic potential in human cancers.

In the present study, we investigated the anti-metastatic activity of daurinol using MDA-MB-231 and A549 cancer cells both *in vitro* and *in vivo*. We observed that daurinol blocked phosphorylation of FAK, which otherwise results in adequate migration, invasion, and expression of uPA, MMP2, and MMP9 and finally increased metastasis *in vitro*. Moreover, oral administration of daurinol inhibited lung metastasis in human breast and lung cancer models (NOD/SCID mice). Furthermore, we found that daurinol inhibited spontaneous tumor formation of NNK/BaP-treated A/J mice. Therefore, our data provide evidence that daurinol may be a preventive anti-metastatic and anti-cancer agent for the treatment of human cancer.

## RESULTS

### Daurinol significantly reduces FAK phosphorylation in human breast and lung cancer cells

**Figure 1:**
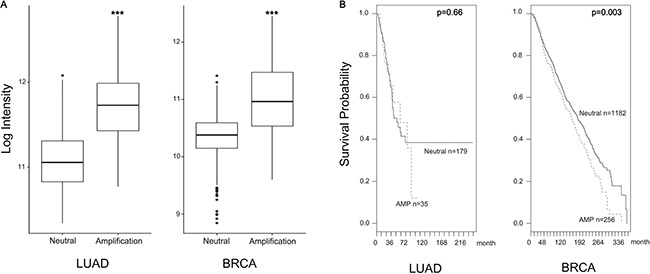
FAK high expression by amplification (**A**) FAK expression increased in the amplification group. FAK mRNA expression values represent mean ± *s.d*., ***p < 0.001 compared with the neutral group. (**B**) FAK amplification was associated with survival probability. Dashed line is FAK amplification group and solid line is FAK neutral group. (Log-rank *p* value in LUAD = 0.66, Log-rank *p* value in BRCA = 0.003).

**Figure 2:**
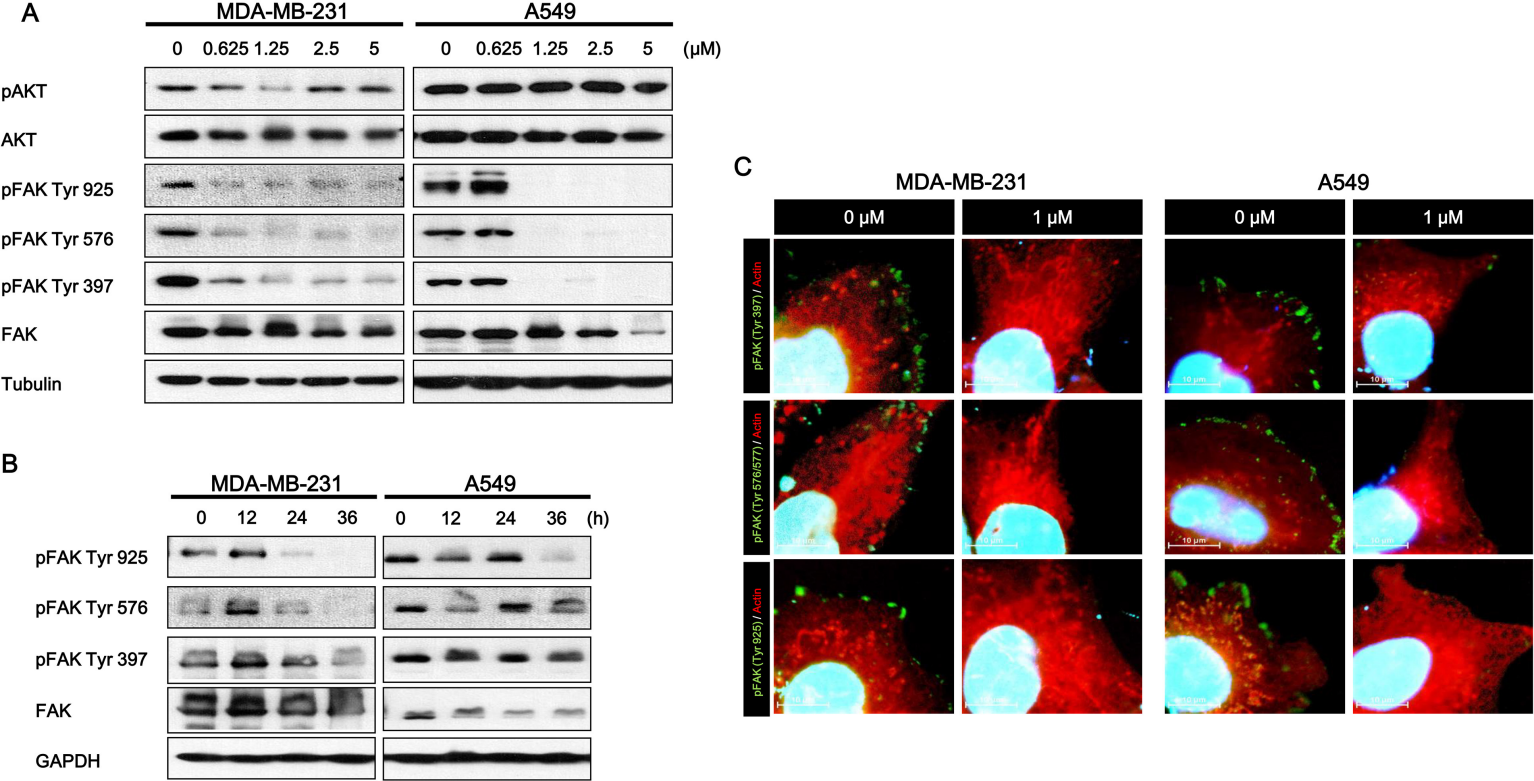
Effect of daurinol on FAK signaling (**A**) Western blotting of total FAK and phosphorylated FAK (Tyr925, Tyr579, and Tyr397) proteins after 48 h of treatment with different concentrations of daurinol in MDA-MB-231 and A549 cancer cells. Cells were lysed, and the indicated proteins were detected by ECL. (**B**) MDA-MB-231 and A549 cancer cells received 1 μM daurinol for indicated times, and cells were lysed, and the indicated proteins were detected by western blotting. Total FAK and phosphorylated FAK (Tyr925, Tyr579, and Tyr397) were detected by ECL. (**C**) Combined staining of phosphorylated FAK (Tyr925, Tyr579, and Tyr397) (stained with Alexa fluor-488) and Actin fiber (stained with TRITC) after daurinol treatment in MDA-MB-231 and A549 cancer cells. Co-staining with DAPI was done to show nuclei.

Focal Adhesion Kinase (FAK), a non-receptor tyrosine protein kinase, is significantly overexpressed and hyper-activated in a majority of solid tumors, including lung and breast cancer [17, 31]. FAK expression is induced by copy number variation, and FAK amplification is associated with patient survival probability. In CNV data, the amplification group was identified based on high level amplification of FAK while the neutral group was identified from samples showing no change in FAK copy number. FAK expression between the amplification and neutral groups was compared by using student's *t*-tests. Log-rank tests and Cox regression analyses were used for survival rate. FAK expression increased in the amplification group (Figure 1A). FAK amplification was also associated with poor prognosis (Figure 1B). As a multifunctional protein, FAK not only regulates extracellular signals from integrin and growth factor receptors but also contributes to cell invasion and metastasis [4, 8, 32]. Therefore, we tested whether or not daurinol inhibits FAK phosphorylation. Western blotting demonstrated that daurinol significantly reduced phosphorylation of FAK Tyr925, Tyr579, and Tyr397 in a dose- and time-dependent manner. Furthermore, we systematically analyzed the potential effects of daurinol on various signaling pathways in A549 and MDA-MB-231 cancer cells. During inhibition of FAK phosphorylation by daurinol only minor modification was detected in pAKT Ser473 under the same experimental conditions (Figure 2A and 2B). The results show that daurinol specifically blocked FAK signaling pathways but not AKT in MDA-MB-231 and A549 cancer cells. Using immunofluorescence staining, we also observed reduction of FAK phosphorylation by daurinol in MDA-MB-231 and A549 cancer cells, respectively (Figure 2D). Taken together, these results provide evidence that daurinol blocks FAK signaling, implying anti-metastasis activity.

### Daurinol inhibits migration and invasion of human breast and lung cancer cells

**Figure 3:**
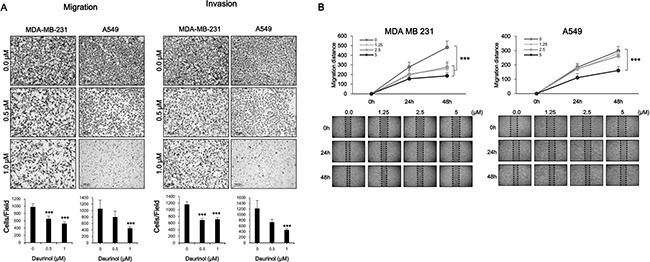
Inhibitory effects of daurinol on migration and invasion (**A**) Migration (left panel) and Invasion (right panel) assays were performed using Boyden's chamber. Indicated concentrations of daurinol treated cells were seeded in the upper chamber of the insert and incubated for 6 h. Images displaying the bottom side of the filter inserts with cells that migrated through the filter pores (upper panel) and columns in the graph represent the analysis of the cell count (bottom panel). (**B**) Monolayers of MDA-MB-231 (left panel) and A549 (right panel) cells were wounded and treated with the indicated concentrations of daurinol. Images of cell migration were captured at 0, 24, and 48 h. Cell motility was determined by measuring the wound closure in six random fields and calculated (upper panels) and Images of wound healing assays (bottom panels). Values represent mean ± *s.d*. of three independent experiments, ***p < 0.001 compared with the untreated control (dose 0).

Tumor metastasis is a dynamic hallmark of cancer, which consists of complicated events; cancer cell migration and invasion are critical steps for cancer metastasis [1, 2]. To investigate the anti-metastasis effect of daurinol, we treated both MDA-MB-231 and A549 cancer cells with different concentrations of daurinol and used trans-well assay. As shown in Figure 3A, 0.5 and 1 μM daurinol treatment reduced the numbers of migrating and invasive MDA-MB-231 and A549 cancer cells in a concentration-dependent manner. The numbers of migrated cells are as follows: MDA-MB-231 with vehicle (979 ± 267.08/field cells), 0.5 μM daurinol (654.5 ± 75.08/field) (compared with vehicle control; *p* < 0.001), or 1 μM daurinol (523 ± 50.90/field) (compared with vehicle control; *p* < 0.001) as well as A549 with vehicle (1057 ± 267.08/field), 0.5 μM daurinol (799 ± 184.67/field) (compared with vehicle control; *p* < 0.07), or 1 μM daurinol (452 ± 94.56/field) (compared with vehicle control; *p* < 0.001). The numbers of invasive cells are as follows: MDA-MB-231 with vehicle (1159 ± 83.43/field cells), 0.5 μM daurinol (687 ± 52.03/field) (compared with vehicle control; *p* < 0.001), or 1 μM daurinol (711 ± 53.39/field) (compared with vehicle control; *p* < 0.001) as well as A549 with vehicle (1217 ± 281.10/field cells), 0.5 μM daurinol (730 ± 103.95/field) (compared with vehicle control; *p* < 0.001), or 1 μM daurinol (456 ± 43.76/field) (compared with vehicle control; *p* < 0.001). This result was further confirmed by wound healing assay to investigate the migration ability of MDA-MB-231 and A549 cancer cells. As shown in Figure 3B, 1.25, 2.5, and 5 μM daurinol treatment reduced the ability of migration from one end of the wound to the other. Migration potentials of both MDA-MB-231 and A549 cancer cells were also significantly inhibited by daurinol treatment. Collectively, these findings suggest that daurinol inhibits migration and invasion potential of lung as well as breast cancer cells at concentrations where no significant inhibition of cell proliferation was observed (Figure 6A).

### Inhibitory effects of daurinol on MMP2, MMP9, and uPA protein activity in human breast and lung cancer cells

**Figure 4:**
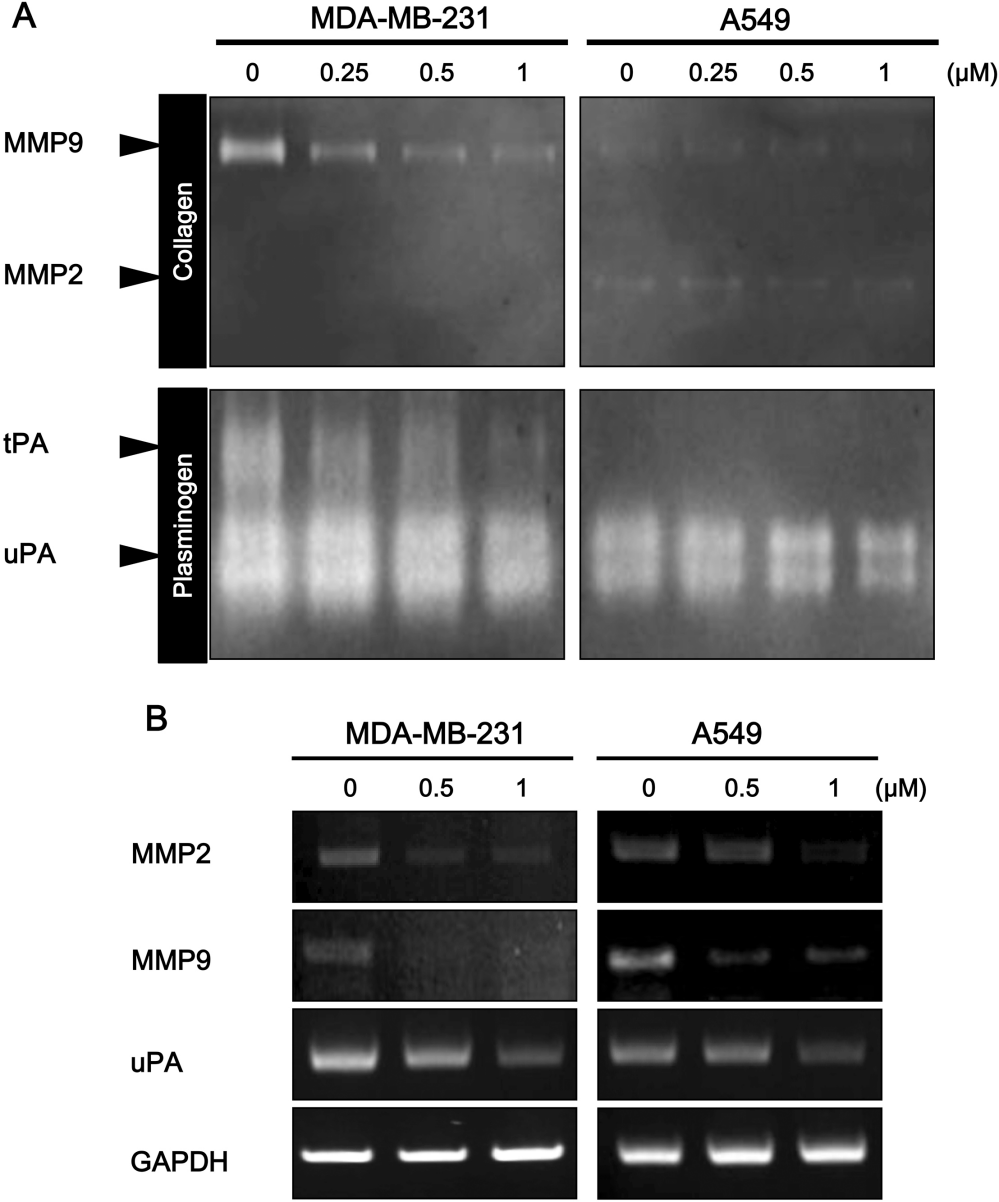
Effect of daurinol on MMP2, MMP9, and uPA expression and activity (**A**) Daurinol-treated MDA-MB-231 and A549 cancer cells CM was subjected to zymograph assay for analysis of MMP2, MMP9, and uPA activities. Equal volumes of serum-free culture medium were obtained from control cells or cells that had been treated with indicated dose of daurinol for 48 hours. Culture media were concentrated with Amicon Ultra-4 centrifugal device obtained from Millipore and loaded onto gels. The proteolytic activity of MMP-2/MMP-9 and uPA was assessed by SDS-PAGE using zymogram gels containing 0.1% (m/v) gelatin and fibrinogen/plasminogen, respectively. (**B**) Semi-quantitative RT-PCR was performed for analysis of MMP2, MMP9, and uPA expression. After 48 h of treatment with different concentrations of daurinol in MDA-MB-231 and A549 cancer cells.

The above results indicate that daurinol could inhibit migration and invasion of human cancer cells. We further determined whether or not inhibition of FAK activity by daurinol is sufficient to suppress expression of MMP2/9 and uPA. Degradation of the ECM surrounding cancer cells is considered as a key event in invasion and metastasis [33]. A previous report found that FAK signalling pathways modulate expression and activation of MMP9, MMP2, and uPA [33–35]. To determine whether or not daurinol can suppress MMP2/9 and uPA expression and activity, we treated cells with daurinol at concentrations of 0.25, 0.5, and 1 μM for 48 h. When CM was analyzed by gelatin and plasminogen zymography, reduced levels of 72/92 kDa (MMP2/MMP9) and 70/45kDa (tPA/uPA) zone clearing activity were detected from daurinol treated MDA-MB-231 and A549 cancer cells compared to the control in a concentration-dependent manner (Figure 4A). RT–PCR analyses revealed lower levels of MMP2, MMP9, and uPA mRNA in daurinol-treated cancer cells (Figure 4B). Together, these results support the conclusion that daurinol mediated FAK inhibition is important in inhibiting cancer cell invasion and that MMP2, MMP9, and uPA expression is regulated by FAK in both MDA-MB-231 and A549 cancer cells.

### Daurinol inhibits metastasis in breast and lung cancer experimental models

**Figure 5:**
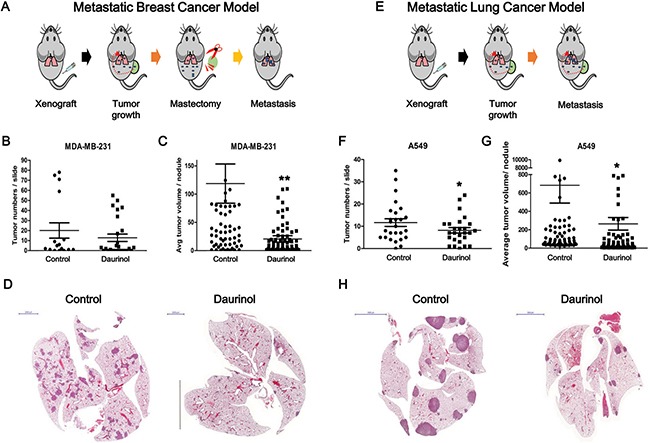
Evaluation of daurinol effect on *in vivo* metastasis Schematic diagram summarizing experimental metastasis approach in breast cancer (**A**) and lung cancer (**E**). (**B**) Number of tumor nodules was quantified per slide. (**C**) Metastatic ability was represented as tumor volumes. (**D**) Representative H&E staining of lung section. (**F**) Number of tumor nodules were quantified per slide. (**G**) Metastatic ability was represented as tumor volumes. (**H**) Representative H&E staining of lung section. Values represent mean ± *s.d*. of three independent experiments, *p < 0.05 and **p < 0.01 compared with the untreated control.

To determine whether or not FAK inhibition is also associated with alteration of *in vivo* tumor metastasis, we designed two different experimental metastasis models. The MDA-MB-231 cell line is derived from the pleural fluid of a patient with ER− metastatic breast cancer [36, 37]. For experimental metastasis assays from bilateral orthotopic inoculations, tumors were extracted from mammary glands up reaching 200 mm^3^ (Figure 5A). Metastatic burden is a combination of both the number of metastases and the emergence of disseminated cells to the lungs after mastectomy. The numbers of tumor nodules are as follows: vehicle control (40.13 ± 63.21/ slide) or daurinol-treated groups (25.62 ± 38.04/field) (compared with vehicle control; no signification). Tumor volumes are as follows: vehicle control (118.69 ± 273.79 μm^3^/tumor) or daurinol-treated groups (22.34 ± 56.20 μm^3^/tumor) (compared with vehicle control; *p* < 0.01) (Figure 5B and 5C). Representative H&E staining of lung sections are shown in Figure 5D. We next used an experimental lung metastasis model to investigate whether or not daurinol inhibits metastasis of lung cancer cells *in vivo*. A549 cells were injected into the right flanks of NOD/SCID mice to generate primary tumors (Figure 5E). Determination of primary tumor volume revealed that tumor growth was not significantly different between the daurinol and vehicle groups. (Data not shown). Neither of the treatment groups exhibited toxicity nor were there any alterations in the body weights of mice. Metastatic burden is a combination of both the number of metastases and size of metastatic tumors. However, when administered 10 mg/kg of daurinol, A549 cells clearly produced less lung nodules and showed significantly reduced metastatic tumor volume in mouse lungs. The numbers of tumor nodules are as follows: vehicle control (23.31 ± 16.62/slide) or daurinol-treated groups (16.38 ± 11.06/field) (compared with vehicle control; *p* < 0.05). Tumor volumes are as follows vehicle control (682 ± 1712.76 μm^3^/tumor) or daurinol-treated groups (264.25 ± 611.98 μm^3^/tumor) (compared with vehicle control; *p* < 0.05) (Figure 5F and 5G). Representative H&E staining of lung sections is shown in Figure 5H. Taken together, our results from both experimental breast and lung metastasis models illustrate that daurinol can inhibit metastasis and survival of human cancer through inhibition of the FAK signaling pathway.

### Inhibitory effects of daurinol on proliferation and survival

**Figure 6:**
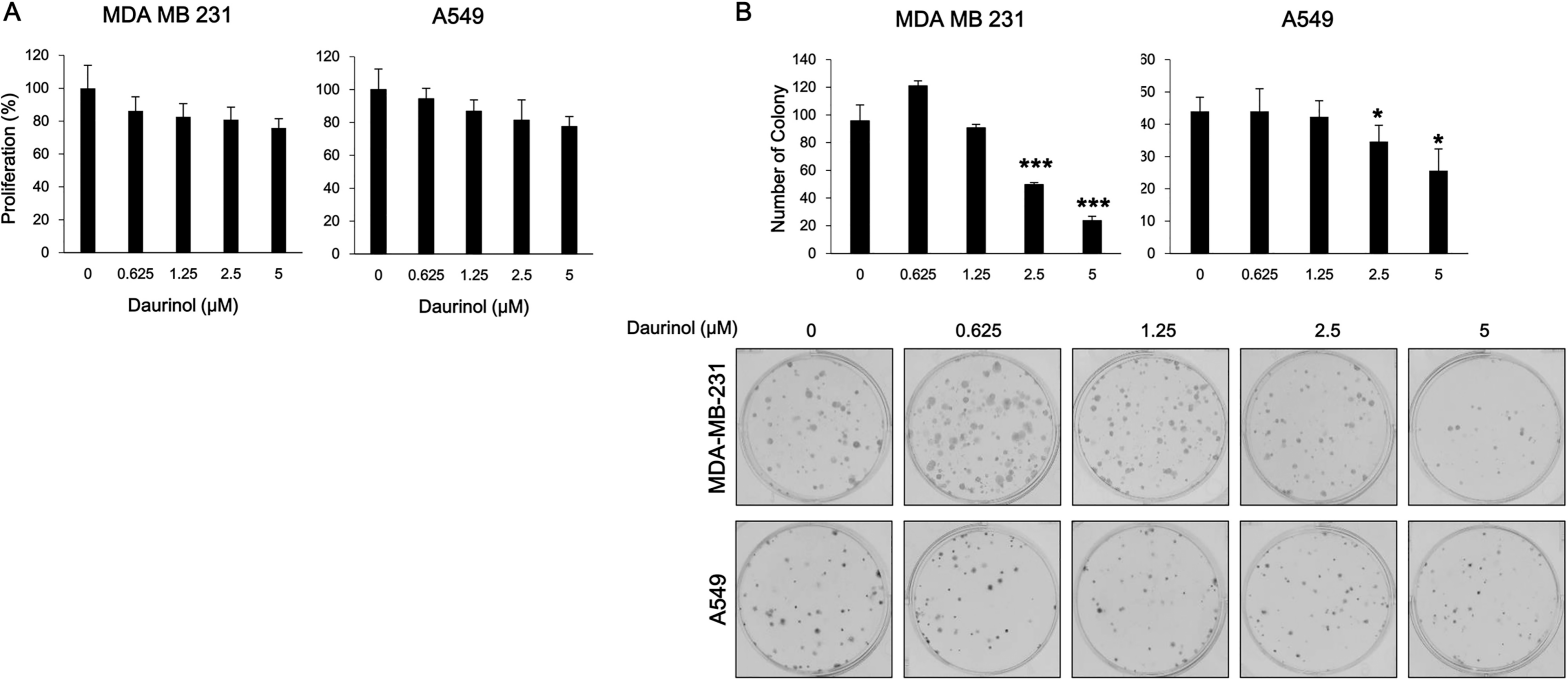
Effects of daurinol on proliferation and survival (**A**) Inhibition of cell proliferation was determined by MTT assay. MDA-MB-231 and A549 cancer cells were treated with daurinol for 48 h. (**B**) Anchorage-dependent colony formation assay was performed as described in Materials and Methods. MDA-MB-231 and A549 cancer cells were treated with daurinol for 10 days and stained with hematoxylin, after which colonies were counted. Values represent mean ± *s.d*. of three independent experiments, *p < 0.05 and ***p < 0.001 compared with the untreated control (dose 0).

The above results indicate that daurinol could inhibit metastasis of human lung and breast cancer cells. At the same time, daurinol suppressed the repopulation of cancer cells in the lung. This suggests that daurinol might inhibit cell proliferation or survival through inhibition of FAK phosphorylation. Cell proliferation assay was performed in order to determine whether or not daurinol could inhibit proliferation. Cell proliferation assay was also performed to determine whether or not the effects of daurinol on cell migration and invasion are due to cytotoxicity. After 48 h of treatment with 0.625, 1.25, 2.5, or 5 μM daurinol, MDA-MB-231 and A549 cells maintained a high level of proliferation potential (Figure 6A). Proliferation rates were 86.25, 82.66, 80.87, or 75.79% (compared with vehicle control; not significant) in MDA-MB-231 cells and 94.43, 86.74, 81.34, or 77.54% (compared with vehicle control; not significant) in A549 cells, respectively. Furthermore, we examined whether or not daurinol could augment inhibition of cell survival by colony formation assay. The colony formation results revealed that colony formation capacity was reduced by daurinol treatment. MDA-MB-231 cells showed a colony formation number of 95.67 ± 11.59/well in the vehicle control as well as number of 121 ± 3.61, 93.57 ± 5.07, 49.67 ± 1.53, or 23.67 ± 3.06/well upon 0.625, 1.25, 2.5, or 5 μM daurinol treatment. On the other hand, A549 cells showed a value of 44 ± 4.36/well in the vehicle control as well as values of 44 ± 7.00, 42.33 ± 4.93, 34.67 ± 5.03, or 25.67 ± 6.66/well upon 0.625, 1.25, 2.5, or 5 μM daurinol treatment (Figure 6B). These results indicate that daurinol inhibits metastasis and repopulation of lung and breast cancer cells via suppression of FAK signaling pathways. In addition, the above results provide evidence that daurinol could be used as a chemopreventive reagent.

### Chemopreventive effect of daurinol on NNK/BaP-treated A/J mouse spontaneous tumor model

**Figure 7:**
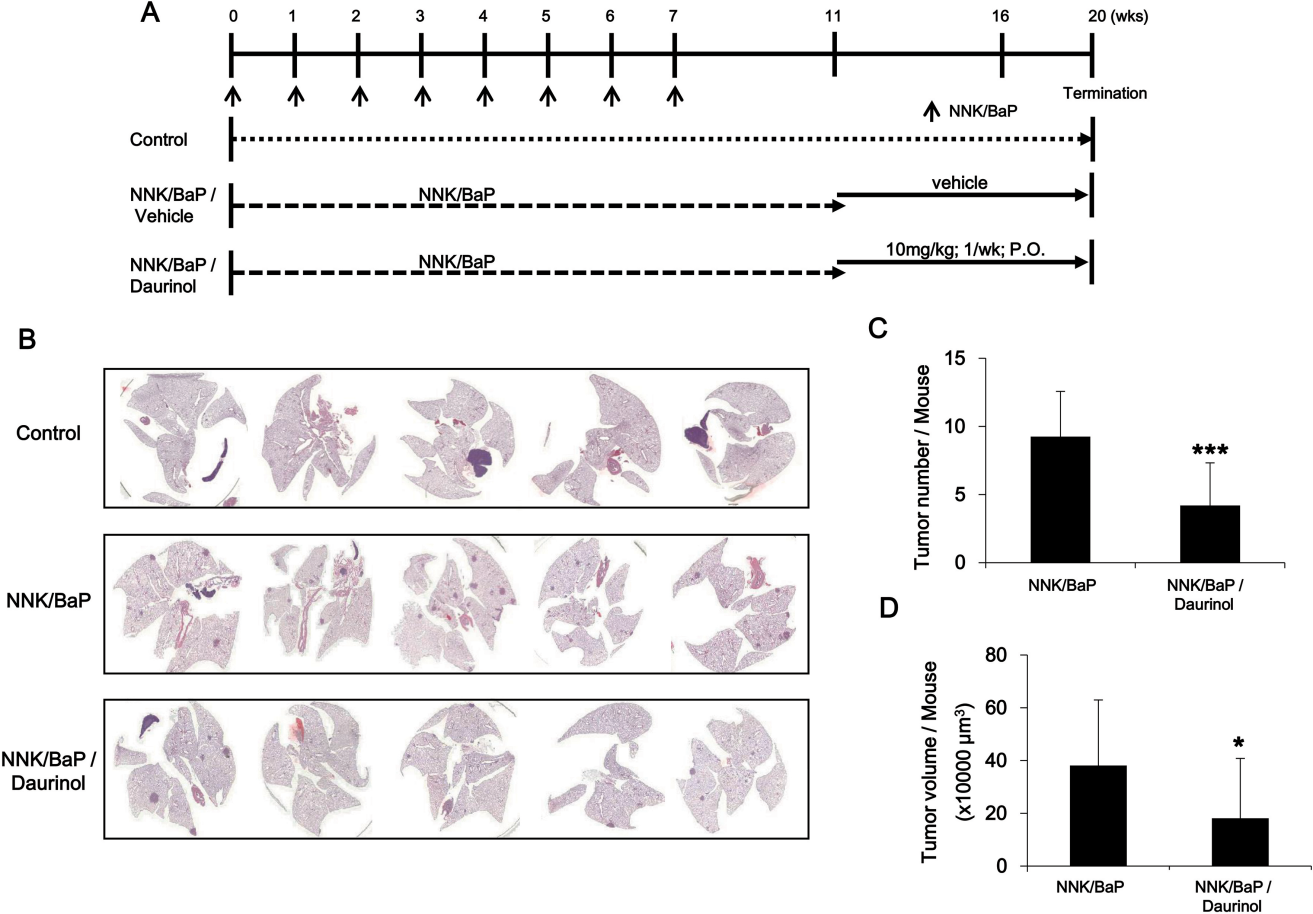
Evaluation of chemopreventive activity of daurinol (**A**) Diagram showing treatment protocol for NNK/BaP and daurinol. (**B**) Representative H&E staining of lung section. (**C**) Numbers of tumor nodules were quantified per mouse. (**D**) Metastatic ability was represented as tumor volumes per mouse. Values represent mean ± *s.d*., *p < 0.05 and ***p < 0.001 compared with the NNK/BaP-treated control (without daurinol).

To assess the chemopreventive activity of daurinol, we used the A/J mouse and NNK/BaP system as described in Materials and Methods and Figure 7A. Animals receiving NNK/BaP (*n* = 10) showed significantly higher tumor incidence and tumor multiplicity compared to control animals. However, daurinol (*n* = 10) significantly reduced tumor number and volume compared to NNK/BaP (Figure 7C and 7D). Daurinol treatment reduced tumor volume as compared to the vehicle control; total tumor volume in daurinol-treated mice was 185.10 ± 125.31 mm^3/^mouse as compared to 382.65 ± 208.83 mm^3/^mouse in the control (*p* < 0.05; Figure 7C), suggesting a chemopreventive effect of daurinol. Tumor number was also notably reduced by NNK/BaP control and daurinol to 9.78 ± 4.23/mouse and 3.60 ± 2.12/mouse, respectively (*p* < 0.001; Figure 7D). These findings indicate not only that daurinol inhibited tumor metastasis but also tumor development.

## DISCUSSION

Recently, many studies reported the importance of natural chemical compounds originating from plant extracts for treatment of human disease [38]. Targeting tumor metastasis using natural products is an efficient therapeutic approach for treatment of metastatic tumors [39–44]. In the present study, we observed that daurinol suppressed metastasis of MDA-MB-231 and A549 cancer cells, and these effects were mediated by FAK signaling inhibition.

Metastatic spread of cancer to bones, lungs, and brain accounts for the majority of cancer-related deaths. Tumor cells require migratory and invasive characteristics to gain access to secondary sites and for successful establishment of a metastatic tumor. Inhibition of this process is an effective therapeutic strategy against cancer metastasis. The development of novel therapeutic agents targeting cell motility is important for preventing tumor metastasis. Daurinol has been reported to have anti-cancer activities such as topoisomerase inhibition and suppression of aurora kinase expression [29, 30]. However, the anti-metastatic effects of daurinol on cancer cells are still unclear. We found that daurinol inhibited cell migration and invasion at minimum cytotoxic concentrations in human breast and lung cancer cells (Figure 2). We also tested whether or not daurinol shows anti-proliferative activity *in vitro*. In MDA-MB-231 and A549 cancer cells, daurinol treatment showed no significant growth inhibitory activity (Figure 6A). These data indicate that daurinol has less cytotoxic effects but possesses significant anti-metastatic properties against breast and lung cancer cells in both *in vitro* assays (Supplementary Figures 1, 2 and 4).

FAK has been reported to be over-expressed in breast and lung cancers, which makes it an attractive target to prevent tumor metastasis [31, 45, 46]. Using a data base, we confirmed that the FAK gene is amplified in a subset of breast and lung cancer specimens. Furthermore, we showed a negative correlation between gene copy number and survival probability in breast and lung cancer patients (Figure 1). In this study, we demonstrated that down-regulation of FAK phosphorylation is involved in daurinol mediated reduction of cancer migration and invasion. Importantly, the current study is the first demonstration that anti-metastatic activity of daurinol is mediated through modulation of FAK activity. FAK inhibitors have been found to reduce metastasis in multiple tumor models [45, 47, 48]. The results from this study indicate that daurinol is a potential anti-metastatic agent in human breast and lung cancer cells with desirable pharmacological properties. In addition, FAK activation further leads to secretion of MMPs such as MMP-2 and MMP-9, which are involved in the degradation of ECM to facilitate cancer cell metastasis. Several reports have shown that inactivation of FAK leads to down-regulation of MMPs and further inhibits tumor cell migration [40, 49]. Daurinol treatment resulted in suppression of MMP2 and MMP9 as well as down-regulation of FAK phosphorylation in both breast and lung cancer cells (Supplementary Figure 3).

Our *in vitro* studies have shown that daurinol has more potent anti-migration/invasion activities than anti-proliferation. The results of our *in vivo* studies are consistent with our *in vitro* data; FAK inhibition by daurinol suppressed breast and lung cancer cell migration and invasion (Figure 5). Our *in vivo* results are also consistent with studies on other tumor types, including melanoma, prostate, pancreas, and glioma cancer, where inhibition of FAK kinase activity results in inhibition of tumor metastasis [50–53]. Effects of FAK kinase inhibition may be attributed to attenuated cell migration, invasion, and lack of tissue remodeling enzymes such as MMP2, MMP9 and uPA, which may impact tumor metastasis.

We have shown that FAK is amplified in breast and lung cancer patient. However, daurinol has not been evaluated regarding its chemopreventive activity. To determine the chemopreventive activity of daurinol, we performed cellular survival assay. In contrast to the anti-proliferative assay, daurinol treatment significantly inhibited colony formation in both cell lines, MDA-MB-231 and A549 (Figure 6B). In agreement with FAK-mediated tumorigenesis, daurinol treatment suppressed chemical-induced spontaneous lung tumors. We speculate that FAK activity regulation in lung cancer may provide the oncogenic signaling required for tumor development in an NNK/BaP-mediated spontaneous lung cancer model (Figure 7). These *in vivo* results are also consistent with studies in other tumor types, including breast, pancreas, and prostate cancer, where inhibition of FAK activity results in modest inhibition of tumor development.

The findings of this study are important since limited therapeutic options are available for highly metastatic breast and lung cancer. Our data demonstrate that daurinol could inhibit invasion, migration, and survival but not proliferation of human cancer cells both *in vivo* and *in vitro*. These inhibitory effects are mediated through inhibition of FAK phosphorylation, leading to reduced MMP2/9 and uPA expression and activation. In addition, daurinol suppressed cell survival *in vitro* and tumor development in an NNK/BaP-induced spontaneous lung cancer model *in vivo*. In general, our observations support therapeutic approaches targeting FAK activity to prevent metastatic tumor spread and tumor development.

## MATERIALS AND METHODS

### High-throughput data analysis

To identify gene expression pattern and clinical association of FAK, high throughput data were used for lung adenocarcinoma (LUAD) and breast cancer (BRCA) samples. All data for gene expression, copy number variation (CNV), and clinical information were downloaded from cBioportal [54]. We selected the LUAD data set for 520 patients and BRCA data set for 2,509 patients [54, 55]. All data for expression and copy number variation were downloaded from TCGA which provide processed data for SNP6.0, microarray and NGS in various cancer types. GISTIC, which is identification tool for genes targeted somatic copy-number alterations from SNP6.0, propose focal amplification event for each patient. Using information of genes based variation in GISTIC, patients were grouped by FAK amplification or neutral. Using RNA-seq and microarray data for same patient samples. FAK expression is induced by copy number variation, and FAK amplification is associated with patient survival probability [56]. The CNV patients in LUAD and BRCA were selected by using copy number level per gene (2: high-level amplification, 0: neutral). We did not select deep loss (-2: possibly homozygous deletion) and shallow loss (-1: possibly heterozygous deletion) samples. We estimated Overall survival rate, because it usually give an overall picture and the survival time for an individual person. FAK expression between amplification and neutral groups was compared by using Student *t*-tests. Log-rank tests and Cox regression analyses were used for survival analyses.

### Western blotting

Western blotting was conducted as previously described [30, 57]. The primary antibodies included pAKT (Ser473), AKT, pFAK (Tyr925), pFAK (Tyr576), pFAK (Tyr397), FAK and tubulin were purchased from Cell Signaling Technology, Inc. (Danvers, MA, USA). The blot was incubated with primary antibodies overnight at 4°C and then horseradish peroxidase-conjugated secondary antibodies for 2 h at room temperature. The protein–antibody complexes were detected using enhanced chemiluminescence (Amersham, Arlington Heights, IL, USA) according to the manufacturer's recommended protocol.

### Semi-quantitative RT-PCR

Total RNA was extracted from control and daurinol treated cells using TRIzol reagent (Ambion, Austin, TX, USA). For semi-quantitative RT-PCR, 1 μg of RNA was used as a template for reverse transcription using the Prime Script 1′st strand cDNA Synthesis kit (Takara; Kyoto, Japan). PCR was carried out with 20 ng of cDNA using a PCR pre-mixture (Bioneer, Daejeon, Korea). The sequences of primers used were as follows: *GAPDH* (forward: 5′-TTTGGTCGTATTGGGCGCCTG-3′; Reverse: 5′-CCATGACGAACATGGGGGCAT-3′), MMP2 (forward: 5′-TCGCCCATCATCAAGTTC-3′; Reverse: 5′-GTGATCTGGTTCTTGTCC-3′), MMP9 (forward: 5′-AACCAATCTCACCGACAG -3′; Reverse: 5′-CAAAGGCGTCGTCAATCA-3′), and uPA (forward: 5′-CCAATTAGGAAGTGTAAGCAGC-3′; Reverse: 5′-GCCAAGAAAGGGACATCTATG-3′). PCR products were separated on 1.5% agarose gel stained with NEOgreen (Insungscience, Seoul, Korea) and visualized under UV lighting using Bio-Rad Gel Doc EZ (Bio-Rad, Hercules, CA, USA).

### Immunofluorescence

For immunofluorescence, human cancer cells were grown on cover slips. After treatment with daurinol for 48 h, cells were rinsed three times with PBS and fixed with Methanol/Acetone (1:1) for 30 minutes at −20°C. Fixed cells were incubated overnight with pFAK (Tyr 925), pFAK (Tyr 576), pFAK (Tyr 397) (Cell signaling Technology, Inc), and Goat anti-Actin (Santa Cruz Biotechnology, Inc., Santa Cruz, CA, USA). Cells were washed three times in PBS containing 0.1 % Triton X-100, followed by incubation with Alexa fluor-488-conjugated goat anti-rabbit IgG (Life Technologies, Carlsbad, CA, USA) and TRITC-conjugated donkey anti-goat IgG (Santa Cruz Biotechnology, Inc) for 2 h. The slides were mounted with Vecta shield mounting medium and viewed under a confocal microscope (Nikon Eclipse Ti, Nikon NY, USA).

### Trans-well migration and invasion

Cell migration and invasion assays were performed using Trans-well Boyden's chamber with an 8.0 μm pore size membrane (BD Biosciences, San Jose, CA, USA). The assay was performed according to the manufacturer's instructions as described by us previously [58]. For the migration and invasion assay, membranes were coated with a 0.5 mg/mL solution of gelatin (Sigma-Aldrich, St. Louis, MO, USA) in distilled water and 0.5 mg/mL solution of matrigel (BD Biosciences, Bedford, MA, USA) in PBS. Daurinol treated cells were seeded in the upper well of Boyden's chamber. The lower chamber was filled with cell culture medium containing 600 μL of medium containing 10% fetal bovine serum (FBS) as a chemoattractant. After 6 h of incubation, non-invaded cells in the upper chamber were removed with a cotton swab. Cells on the bottom side were fixed in 100% methanol and stained with hematoxylin. The invaded cells were imaged using a bright field microscope (Olympus Inc., Tokyo, Japan). The cells were quantified using ImageJ program. Each experiment was conducted at least twice with three sets per experiment. The average values of representative results were graphed.

### Wound healing assay

Wound-healing assay was performed as described previously [58]. Briefly, MDA-MB-231 and A549 cells were incubated to form a monolayer in 6-well dishes. Wound was created by scratching the monolayer with a 1 mL pipet tip. Cells were incubated with the desired concentration of daurinol. The wound was imaged at the desired time point (24 and 48 h) using a bright field microscope (Olympus Inc., Tokyo, Japan). Wound widths were quantified using ImageJ program. Each experiment was conducted at least twice with three sets per experiment. The average values of representative results were graphed.

### Cell proliferation and colony formation

Human breast cancer cell line MDA-MB-231 was purchased from ATCC. Luciferase-labeled human lung cancer cell line A549 was purchased from Caliper (Hopkinton, MA, USA). The cell lines have not been subsequently authenticated since being received. These cell lines were cultured in RPMI 1640 (Welgene, Daegu, Korea) supplemented with 10% fetal bovine serum (FBS; Welgene) and 1% penicillin–streptomycin (GIBCO-BRL Life Technologies; Gaithersburg, MD, USA). All cell lines were cultured at 37°C in the presence of 5% CO_2_. Cell proliferation was measured using 3-(4,5-dimethylthiazol-2-yl)-2,5-diphenyltetrazolium bromide (MTT) assay as described previously [30]. Cells were plated at a density of about 3,000 cells/well in 96-well plates and incubated overnight. Cells were then treated with different concentrations of daurinol. After desired duration of treatment (48 h), 10 μl of 5 mg/ml MTT (purchased from Sigma Aldrich, St. Louis, MO, USA) was added to each well and incubated for 2 h at 37°C. Absorbance was measured at 590 nM. Inhibition of cell proliferation by daurinol was calculated based on the absorbance ratio between treatment and control. For the anchorage-dependent colony formation assay, cancer cells were seeded into 6-well culture plates at a density of 5 × 10^2^ cells/well. After overnight incubation, cells were treated with different concentrations of daurinol and maintained for 7 days at 37°C. Finally, plates were stained with hematoxylin, and the colony number was counted.

### Zymogram

The proteolytic activities of MMP-2, MMP-9, and uPA in CM (conditioned medium) were analyzed by substrate-gel electrophoresis using SDS-PAGE gels containing 0.2% (m/v) gelatin or 0.12% (m/v) fibrinogen and plasminogen (0.01 NIH unit/mL) [59, 60]. CM from each control and the daurinol treatment group was concentrated using an Amicon Ultra-4 centrifugal device (Millipore, Bedford, MA, USA), and 5 μg of each sample was loaded onto gels. After electrophoresis, gels were washed with 2.5% Triton X-100 and incubated overnight in zymogram incubation buffer (50 mM Tris-HCl, 0.15 M NaCl, 10 mM CaCl_2_, and 0.02% NaN_3_) at 37°C. Clear bands indicative of enzymatic activity were visualized by staining gels with coomassie blue.

### *In vivo* animal study

Investigation was conducted in accordance with the ethical standards and according to the approved protocol by the College of Pharmacy, Gachon University and National Cancer Center, Institutional Animal Care and Use Committee (IACUC).

Experimental lung cancer metastasis model was established as described previously [58]. In brief, 6-week-old male NOD/SCID mice ((NOD/LtSz-Prkdcscid/J) KRIBB, Cheongwon-gun, Korea) were inoculated by A549 human lung cancer cells into the right flank. When the tumor was grown and reached the standard volume of approximately 200 mm^3^, mice were randomly assigned to two experimental groups (*n* = 10) and treated with daurinol (10 mg/kg of body weight) or vehicle by oral gavage twice a week for 6 weeks. For the experimental breast cancer metastasis model, 6-week-old female NOD/SCID mice were used. MDA-MB-231 human breast cancer cells were orthotopically transplanted into the mammary fat pads of mice, as described previously [61–63]. Once recipient mammary tumors reached a size of approximately 200 mm^3^, a mastectomy was conducted. After induction of anesthesia and disinfection, a 2 cm midline abdominal skin incision was made and tumor supplying arteries were located and ligated. The mammary tumor including adjacent fourth and fifth mammary glands were separated from adherent tissues using forceps and soaked cotton swabs. The skin was closed using stitches. After resection, mice were treated with oral gavage injection of daurinol (10 mg/kg of body weight) or vehicle twice a week for 6 weeks. Following mastectomy, all mice were monitored for disease progression and metastasis formation by palpation and daily observation of their physical health, appearance, and behavior. Recipient animals were sacrificed when they developed clinical signs of distress caused by metastatic disease. After treatment, all mice were sacrificed, after which the lungs were removed and fixed in 10% formalin. The tissue was submitted to histological evaluation using hematoxilin and eosin (H&E) staining, and tumor number and volume quantified. The tumor volume was calculated by the formula: μm^3^ = 1/2 × long diameter × short diameter^2^.

To assess the chemopreventive effect of daurinol on tobacco-induced tumorigenesis, 6-week-old female A/J mice (from Orient Co. (Seoul, Korea)) were treated with 4-(methylnitrosoamino)-1-(3-pyridyl)-1-butanone (NNK) and benzo(a)pyrene [BaP] (3 μM each in 0.1 mL of cottonseed oil) once a week for 8 weeks. After the first treatment, mice were randomly divided into two groups. One group of mice received vehicle alone while the second group received 10 mg/kg of daurinol. Daurinol was administered once a week for 10 weeks. After treatment, all mice were sacrificed, after which lungs were removed and fixed in 10% formalin. Tissues were submitted to histological evaluation using hematoxilin and eosin (H&E) staining, and number and volumewere quantified. Tumor volume was calculated by the formula: mm^3^ = 1/2 × long diameter × short diameter^2^.

### Statistical analysis

Two-tailed Student's *t*-test was used for statistical analysis of comparative data using Microsoft Excel software (Microsoft Co., Tokyo, Japan). Values of *p* < 0.05 were considered as significant and indicated by asterisks in the figures. For graphical representation of data, y-axis error bars indicate standard deviation (*s.d*.) of the data for each point marked on the graph.

## SUPPLEMENTARY MATERIALS FIGURES


