# Supplementary material for: Daurinol blocks breast and lung cancer metastasis and development by inhibition of focal adhesion kinase (FAK)
Source: Oncotarget. 2017 Jul 4;8(34):57058–71. doi: 10.18632/oncotarget.18983 (PMC5593625; doi:10.18632/oncotarget.18983)
Supplement: Supplementary file 1 [file oncotarget-08-57058-s001.pdf]

Daurinol blocks breast and lung cancer metastasis and development by inhibition of focal adhesion kinase (FAK)

SUPPLEMENTARY MATERIALS

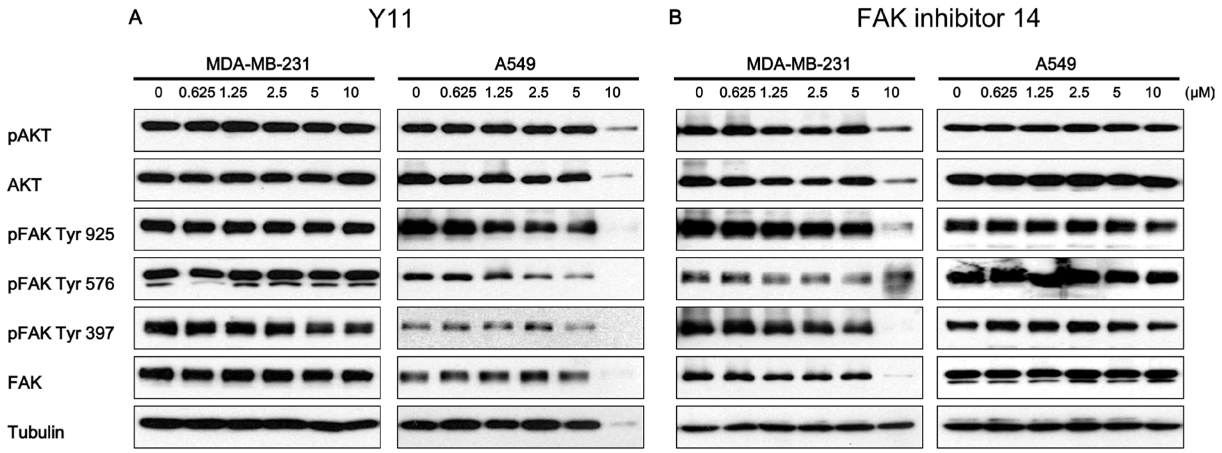

Supplementary Figure 1: Effect of FAK inhibitors on FAK signaling. Western blotting of total FAK and phosphorylated FAK proteins after 48 h of treatment with different concentrations of FAK inhibitor Y11 (**A**) or 14 (**B**) in MDA-MB-231 and A549 cancer cells. Western blotting analysis for Total FAK, phosphorylated FAK (Tyr925, Tyr579, and Tyr397), Total AKT, and phosphorylated AKT (Ser473). Tubulin were served as a standard for sample normalization.

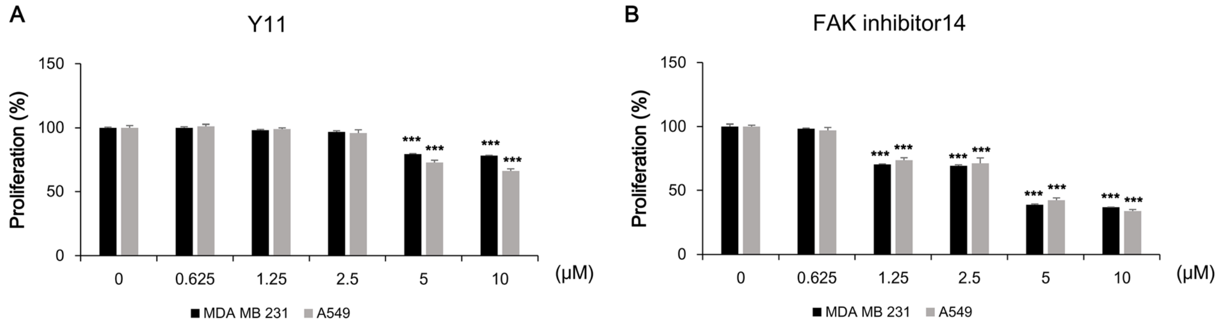

Supplementary Figure 2: Effects of FAK inhibitors on proliferation. Inhibition of cell proliferation was determined by MTT assay. MDA-MB-231 and A549 cancer cells were treated with FAK inhibitor Y11 (**A**) or 14 (**B**) for 48 h. Values represent mean ± s.d. of three independent experiments, ***$p < 0.001$ compared with the untreated control (dose 0).

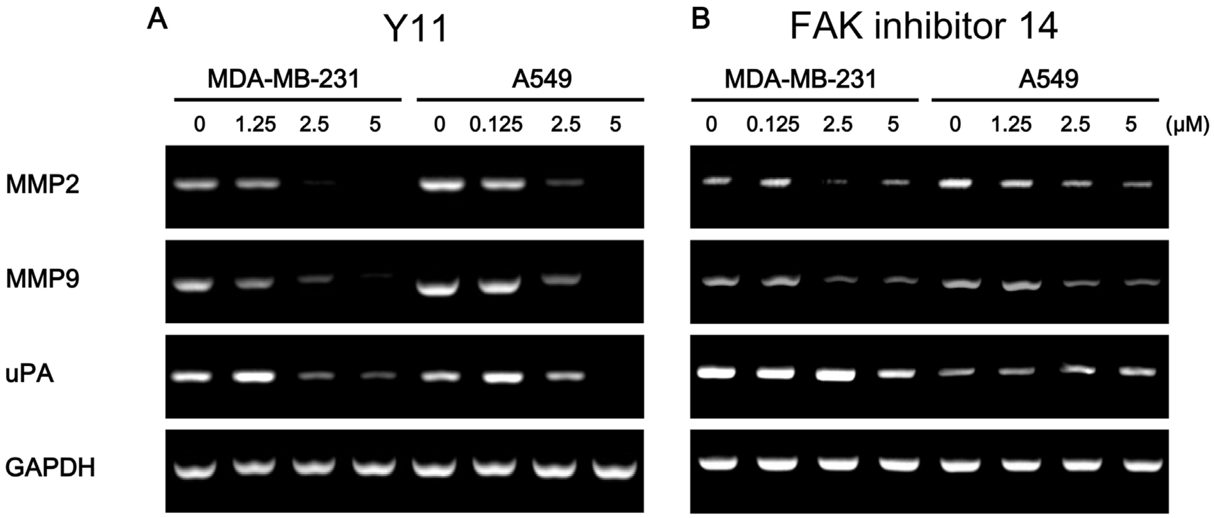

Supplementary Figure 3: Effect of FAK inhibitors on MMP2, MMP9, and uPA mRNA expression. Gene expression patterns of MMP2, MMP9, and uPA in MDA-MB-231 and A549 cancer cells under FAK inhibitor Y11 (**A**) or 14 (**B**) treatment were evaluated by semi-quantitative RT-PCR. The MDA-MB-231 and A549 cancer cells were incubated with indicated concentration of FAK inhibitor Y11 or 14 for 48 h. A control PCR was also done for GAPDH which was served as a standard for sample normalization.

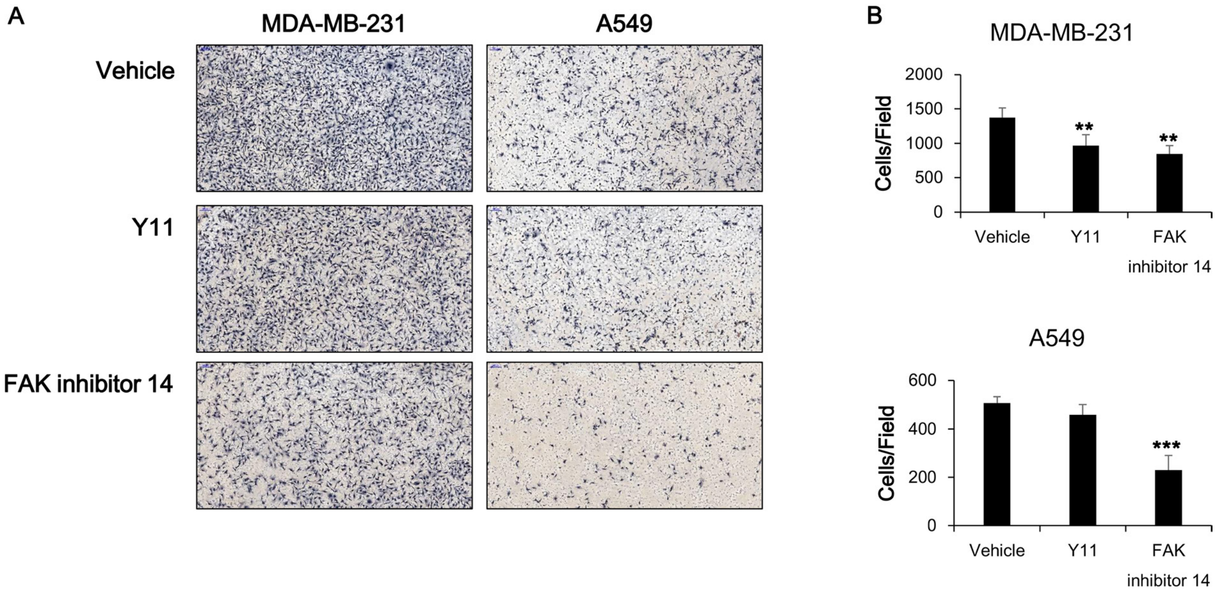

Supplementary Figure 4: Inhibitory effects of FAK inhibitors on migration. Migration assays were performed using Boyden's chamber. 1 μ M of FAK inhibitor Y11 or 14 treated MDA-MB-231 and A549 cancer cells were seeded in the upper chamber of the insert and incubated for 6 h. Representative pictures (**A**) and Invasion (right panel) assays were performed. (**B**)Values represent mean ± s.d. of three independent experiments, ***$p < 0.001$ compared with the untreated control (dose 0).
